# Authors’ Reply: Involving Health, Technology, and Financial Stakeholders in Co-Designing Digital Pathways for Value-Based Care

**DOI:** 10.2196/86837

**Published:** 2025-12-04

**Authors:** Jinsong Chen, Christopher Bullen, Lan Zhang

**Affiliations:** 1 University of Auckland, Auckland, New Zealand; 2 Zhejiang University, Hangzhou, China; 3 Hangzhou City University, 51 Huzhou Street, Gongshu District, Hangzhou, China, 86 13588709515

**Keywords:** digital health, value-based health care, VBHC, patient-reported outcome measures, PROM, digital transformation, health care innovation, patient-centric care, health technology, patient-reported outcome, PRO, outcome measure, telehealth, telemedicine, eHealth, personalized, customized, engagement, patient-centered care, standardization, implementation

We appreciate the thoughtful comments shared in response to our viewpoint, “Digital Health Innovations to Catalyze the Transition to Value-Based Health Care” [1]. The correspondents have highlighted several critical dimensions that must be addressed for value-based health care (VBHC) to move from theory to real-world practice [2].

We agree that realizing VBHC requires much more than the adoption of digital technologies—it calls for a systemic reorientation of how health systems deliver, measure, and fund care. The points raised regarding patient co-design, digital equity, data interoperability, and integration into workflows align strongly with our original arguments [1]. As we noted, digital transformation must go beyond digitization and instead support participatory, patient-centered models that meaningfully engage patients and clinicians in decision-making processes.

We also concur that trust is foundational to successful adoption. As health systems increasingly rely on patient-reported outcome measures (PROMs) and patient-reported experience measures, transparent governance and ethical data practices will be key to building confidence among both patients and providers. Similar to prior recommendations in the literature [3], we view trust-building as integral to ensuring that patient-generated data are used responsibly to inform care decisions and improve outcomes.

The correspondents’ emphasis on education and training for health care professionals is also well taken. As we continue to study digital PROM implementation in clinical settings, we are exploring structured capacity-building initiatives to strengthen clinicians’ digital literacy and analytic skills—ensuring they can interpret and act upon PROM and patient-reported experience measure data in a timely, patient-centered manner.

Moreover, we appreciate the thoughtful expansion on sustainable financing and business models. We fully agree that aligning payment systems with outcomes is central to sustaining VBHC reforms. As highlighted in recent research on alternative payment models [4,5], outcome-linked incentives and cross-sector collaboration are essential to support the infrastructure, workforce, and long-term viability of value-based initiatives.

In conclusion, we share the correspondents’ vision for a holistic and equitable transformation of health systems. Realizing the promise of digital health for VBHC requires concurrent progress in technology, governance, education, and financing. We echo their call for further empirical research across diverse health system contexts to identify actionable pathways for scaling digital innovations that deliver measurable value for patients, providers, and society.

## Abbreviations

PROM: patient-reported outcome measure
VBHC: value-based health care
